# Reduced prevalence of soil-transmitted helminths and high frequency of protozoan infections in the surrounding urban area of Curitiba, Paraná, Brazil

**DOI:** 10.1016/j.parepi.2019.e00115

**Published:** 2019-07-31

**Authors:** Camila Yumi Oishi, Débora do Rocio Klisiowicz, Raimundo Seguí, Pamela C. Köster, David Carmena, Rafael Toledo, José Guillermo Esteban, Carla Muñoz-Antoli

**Affiliations:** a Post-Graduate Program in Microbiology and Pathology, Department of Basic Pathology, Biological Sciences Area, Paraná Federal University, Curitiba, Brazil; b Departamento Farmacia y Tecnología Farmacéutica y Parasitología, Área Parasitología, Facultat Farmàcia, Universitat València, Spain; c Parasitology Reference and Research Laboratory, National Centre for Microbiology, Majadahonda, Madrid, Spain

**Keywords:** Schoolchildren, Soil-transmitted helminths, *Blastocystis*, Curitiba, Paraná, Brazil

## Abstract

Human populations living in the surrounding urban areas of large Brazilian cities have increased vulnerability to intestinal parasites. However, the epidemiological scenario of soil-transmitted helminths (STH) in Curitiba, Paraná’s main city, remains largely unknown. To bridge this gap of knowledge, this study aims to determine the prevalence of intestinal parasites and to investigate potential transmission pathways of the most prevalent species detected. We conducted a cross-sectional epidemiological study between July and September 2014 among schoolchildren in urban and peri-urban (deprived) areas of the municipality of Campo do Tenente, Curitiba. A total of 549 stool samples were used for coproparasitological diagnosis. Microscopy-positive samples of the most common species found were re-assessed by PCR and sequencing methods at the small subunit rRNA gene. Prevalence of infection by any given enteroparasite was 24.8%, but soil-transmitted helminths were only detected in 3.5% of the examined samples. Frequency of protozoan infections reached 90% and 97.8% in single and multiple infections, respectively. *Blastocystis* sp. (38.9%) was the most frequently species found in the surveyed schoolchildren population. A total of 41 *Blastocystis*-positive samples were unambiguously typed as ST1 (36.4%), ST2 (21.2%), ST3 (39.4%), and ST1 + ST3 mixed infection (3.0%). These results indicate that *Blastocystis* transmission is primarily anthroponotic in origin. This data highlights the importance of maintaining the anthelminthic control programs currently in place and of improving sanitary disposal of human excreta in poor-resource settings.

## 1 Introduction

Infections by intestinal protozoan and helminth species are among the most prevalent parasitic diseases in tropical and subtropical developing countries, where they constitute a major public health issue and socio-economic concern (WHO, 2017). About 3.5 billion people are infected by intestinal parasites globally. Young children living in poor-resource settings are particularly at risk, as these pathogens may impair their cognitive and psychomotor development (Crompton and Nesheim, 2002; Mbuh and Nembu, 2013). Epidemiological surveys targeting different human populations are highly needed in developing countries to estimate the actual status of intestinal parasitic infections in order to allow the implementation of appropriate control measures, or to evaluate the impact of ongoing programs for parasite control.

In several parts of the world, soil-transmitted helminths (STH) are experiencing a significant reduction in prevalence rates (Periago et al., 2018). However, beside STH infections, protozoan disease outbreaks are being increasingly reported. Transmission typically follows the faecal-oral route, either directly (e.g. person-to person or animal-to-person contact) or indirectly through ingestion of contaminated food, water or fomites (Corrales et al., 2006). In this regard, preventive and control measures against cryptosporidiosis, giardiasis or even blastocystosis should be emphasized (Henriques Coelho et al., 2017; Valença Barbosa et al., 2017).

Epidemiological surveys carried out globally have reported the Stramenopile *Blastocystis* sp. as the most common eukaryotic organism in human faecal samples (Souppart et al., 2009). The role of *Blastocystis* as a causative agent of diarrhoea and other intestinal or extra-intestinal disorders remains controversial because this parasite species can be found in both symptomatic and asymptomatic individuals (Stensvold et al., 2009). The high genetic diversity within *Blastocystis* has led to the appearance of numerous molecular epidemiological studies aiming to ascertain potential sources of infection and transmission pathways. In Brazil, since the initial description of *Blastocystis* subtypes (STs) circulating in Amazonian indigenous populations (Malheiros et al., 2011), several surveys have been recently published on this topic (Valença Barbosa et al., 2017, Valença Barbosa et al., 2018; Oliveira-Arbex et al., 2018; Seguí et al., 2018).

Curitiba is the main city of Paraná state in southern Brazil. The occurrence of STH contamination in Curitiba’s public parks and squares has been previously described (Sprenger et al., 2014). Other epidemiological studies on the presence of intestinal parasites in Paraná state have been carried out in children from the remote area of Guarapuava (Buschini et al., 2007), and in community surveys conducted in Pitanga (Nascimento and Moitinho, 2005) and the Paranaguá bay (Seguí et al., 2017, Seguí et al., 2018). Based on its motor industry, Curitiba has experienced uninterrupted economic growth in recent years, although serious urban and environmental problems including inadequate water supplies, surface water pollution, and landfill depletion, still persist. A clear example of this situation is the growing municipality of Campo do Tenente, an important centre in the ancient path of livestock transportation from Uruguay.

This study was specifically designed to evaluate: i) the prevalence and frequency of intestinal parasites in the schoolchildren population from the surrounding urban area of Curitiba, and their distribution according to sex and age; ii) the occurrence of single and multiple infections; and iii) potential transmission pathways of the most prevalent species detected. Molecular epidemiological information generated here is expected to expand our current knowledge on the parasitological scenario of these pathogens in large Brazilian cities, enabling direct comparison of genotyping data, with those previously reported in the country.

## 2 Material and methods

### 2.1 Sampling

The municipality of Campo do Tenente is <80 km away from the metropolitan area of Curitiba, the capital of Paraná in the southern region of Brazil. It covers 302 km^2^, encompassing an urban area of 50 km^2^ and a rural area of 252 km^2^, respectively. The municipality limits north-west with Lapa, north-east with Quitandinha, east with Piên and south-west with Rio Negro municipalities. The region has a humid, subtropical climate with an average annual temperature of 17 °C. The total population is about 7125 inhabitants, of which 58.9% live in urban areas and 41.1% in rural areas, respectively. The population density is near seven times greater in urban (83.8 inhabitants/km^2^) than in rural (11.6 inhabitants/km^2^) areas (ITCG, 2017). The public schools in Campo do Tenente provides education to 1774 schoolchildren (INEP, 2015).

A cross-sectional epidemiological study was conducted between July and September 2014.

The minimum sample size (n) was estimated at 315 schoolchildren using the formulae

$$n=\frac{{\mathrm{N\alpha}}^{2}Z^{2}}{\left(N-1\right)e^{2}+\gamma^{2}Z^{2}}$$

where N is the total population to be analysed (1774 schoolchildren according to INEP, 2015), α is the sample standard deviation (set at 0.5), Z is the statistic corresponding to the level of confidence (for a 95% level of confidence the fixed value was 1.96), e is the marginal error (set at 5%), and γ is the expected prevalence (set at 50% based on the intestinal parasite prevalence rate previously reported in Brazil by Chammartin et al., 2013).

The municipality of Campo do Tenente (Curitiba) encompassed the Centro downtown (a developed administrative and commercial area with paved streets) and the Divino zone (a peri-urban economically depressed area only 1 km away from downtown). Samples were obtained from 12 randomly selected public schools. The participation was always on a voluntary basis. Pre-labelled sampling kits including instructions on how to collect the stool sample safely were employed. All the kits were distributed among participating schools. Basic socio-demographic data (age and gender) from each participant was obtained at the time of the school visit, and stool samples were collected in the following days. In all cases a single stool sample was obtained per participant. Collected stool samples were kept refrigerated and transported to the Department of Basic Pathology, Biological Sciences Area, Paraná Federal University (Curitiba, Brazil) for processing and analysis.

### 2.2 Stool sample processing

Stool samples were homogenized and fixed with 10% formalin in a 1:3 proportion and used for coproparasitological diagnosis by the modified Ritchie concentration technique (Knight et al., 1976). Concentrates obtained were also used to produce faecal smears stained by the modified Ziehl-Neelsen method to detect *Cryptosporidium* spp. oocysts. A small aliquot of each unpreserved stool sample was placed in a vial containing 70% ethanol and shipped to the Valencia University (Valencia, Spain) for downstream molecular analyses.

Prevalence rates and frequency of parasite distribution were calculated for each parasite species found. For information purposes, diagnostic results were sent to each participating individual and reported to the Unidades de Saúde do Campo do Tenente in order to initiate appropriate treatments, if needed.

### 2.3 DNA extraction

Total DNA was extracted from 200 mg of concentrated faecal material using QIAamp DNA Stool Mini Kit (Qiagen, Hilden, Germany), following the manufacturer’s instructions. Purified DNA samples (200 μL) were stored at −20 °C until use.

### 2.4 Molecular characterization of *Blastocystis* sp.

Only *Blastocystis*-positive samples at microscopy examination were subsequently used for molecular characterization analyses. A direct PCR protocol was performed using the pan-*Blastocystis* barcode primers RD5 (5′-ATCTGGTTGATCCTGCCAGT-3′) and BhRDr (5′-GAGCTTTTTAACTGCAACAACG-3′) to amplify a partial fragment (~600 bp) of the gene coding for the small subunit (*SSU*) rRNA of the eukaryote (Scicluna et al., 2006). Amplification reactions (25 μL) included 2.5 units of MyTAQ™ DNA polymerase (Bioline GmbH, Luckenwalde, Germany), 5xMyTAQ reaction buffer containing 5 mM dNTPs and 15 mM MgCl_2_, 5 μL of template DNA and 0.5 μM of the primer set RD5/BhRDr. The amplification protocol (one step of 95 °C for 3 min, followed by 30 cycles of 1 min each at 94, 59, and 72 °C, with an additional 2 min final extension at 72 °C) was conducted on a 2720 thermal cycler (Applied Biosystems, CA, USA). Obtained amplicons were visualized on 2% agarose gels stained with Pronasafe nucleic acid staining solution (Conda, Madrid, Spain).

Positive-PCR products were sequenced in both directions using the primer set described above. DNA sequencing was conducted using BigDye Terminator chemistry (Applied Biosystems) on an ABI PRISM 3130 automated DNA sequencer.

### 2.5 Sequence analyses

Raw sequencing data in both forward and reverse directions were viewed using Chromas Lite version 2.1 sequence analysis program (http://chromaslite.software.informer.com/2.1/), and the MEGA 6 free software (http://www.megasoftware.net/) was used to align the obtained sequences. Generated consensus sequences were submitted to the BLAST tool (http://blast.ncbi.nlm.nih.gov/Blast.cgi) to confirm the presence of *Blastocystis* sp. *Blastocystis* sequences were then submitted to the *Blastocystis* 18S database (http://pubmlst.org/*Blastocystis*/) for sub-type confirmation and allele identification. The sequences obtained in this study have been deposited in GenBank under accession numbers MH493729 to MH493737.

### 2.6 Data analyses

Data were analysed with the free software Open Epi version 3.01. The chi-square test was used to compare infection rates according to area, sex and age group. A probability of p < 0.05 was considered statistically significant.

### 2.7 Ethics

The study design and procedures involved during recruitment and sample collection have been approved by the Comitê de Ética em Pesquisa da Universidade Federal do Paraná (CAAE 09152012.3.0000.0102). Every school participant was provided with detailed information and study protocols explaining the goals of the survey and how to participate in it. A signed informed consent was obtained from the parent/legal guardian of each schoolchildren that voluntary participated in the study.

## 3 Results

### 3.1 Study population and intestinal parasite survey

A total of 549 stool samples were collected, including 267 (48.6%) from boys and 282 (51.4%) from girls. These figures represented approximately 30% of the total schoolchildren population in the surveyed area, exceeding the sample size initially estimated.

Total prevalence of infection was 24.8% (136/549) (Table 1). Total protozoan prevalence (22.9%, 126/549) was significantly higher (p < 0.001) than that of STHs (3.5%, 19/549). Overall, *Blastocystis* sp. (8.9%, 49/549) was the most prevalent parasite species identified, with *A. lumbricoides* (2.4%, 13/549) being the most prevalent STH detected (Table 1). Of note, no coccidian infections were observed after examination of Ziehl-Neelsen stained faecal smears.

**Table 1:** Prevalence of infection by intestinal parasites in the schoolchildren population from the surrounding urban area of Curitiba (Brazil) (N = total participants; n = number of infected; % = percentage; 95% CI = 95% confidence interval).

	Surrounding urban area of Curitiba		
	N = 549		
	n	%	95% CI
Protozoa	126	22.9	16.6–26.6
*Entamoeba coli*	41	7.5	5.5–9.9
*Entamoeba histolytica*/*dispar*	11	2.0	1.1–3.5
*Entamoeba hartmanni*	21	3.8	2.4–5.7
*Endolimax nana*	22	4.0	2.6–5.9
*Iodamoeba buetchli*	6	1.1	0.4–2.3
*Giardia intestinalis*	29	5.3	3.6–7.4
*Chilomastix mesnili*	5	0.9	0.3–2.0
Stramenopiles			
*Blastocystis* sp.	49	8.9	6.7–11.5
Helminths	19	3.5	2.2–5.3
*Ascaris lumbricoides*	13	2.4	1.3–3.9
*Trichuris trichiura*	5	0.9	0.3–2.0
*Strongyloides stercoralis*	1	0.2	0.1–0.8
*Enterobius vermicularis*	3	0.5	0.1–1.5
Total infected	136	24.8	21.3–28.5
Negative	413	75.2	71.5–78.7

Parasite infections were distributed between boys (25.1%, 67/267) and girls (24.5%, 69/282) with no statistical differences (p = 0.943) (Table 2). Sex was found to be a risk factor neither for protozoan nor STHs infections. Schoolchildren aged 0–4 years were more vulnerable to infections by intestinal parasites (31.1%, 14/45) than those belonging to other age groups, but without reaching statistical significance (p = 0.644) (Table 2).

**Table 2:** Prevalence of protozoa and helminths infection in schoolchildren from the surrounding urban area of Curitiba (Brazil) according to sex and age group (N = total participants; n = number of infected; % = percentage; 95% CI = 95% confidence interval; p-value = statistical value).

	N	Surrounding urban area of Curitiba											
		Total infected				Protozoa				Helminths			
		n	%	95% CI	p-Value	n	%	95% CI	p-Value	n	%	95% CI	p-Value
Sex													
Boys	267	67	25.1	20.2–30.6	0.943	63	23.6	18.8–28.9	0.804	9	3.4	1.7–6.1	0.903
Girls	282	69	24.5	19.7–29.7		63	22.3	17.8–27.5		10	3.5	1.8–6.2	
Age group													
0–4	45	14	31.1	18.9–45.7	0.644	13	28.9	17.1–43.3	0.401	1	2.2	0.1–10.5	0.906
5–9	339	83	24.5	20.1–29.3		78	23.0	18.8–22.7		11	3.2	1.7–5.6	
10–14	119	30	25.2	18.1–33.6		28	23.5	16.6–31.8		5	4.2	1.6–9.1	
≥15	46	9	19.6	9.9–32.9		7	15.2	6.9–27.8		2	4.3	0.7–13.6	

Single infections (66.2%, 90/136) were significantly (p < 0.001) more frequent than any given multiple-infection combination (33.8%, 46/136) (Table 3). Frequency of protozoan infections reached the highest values compared to STHs, either in single (90%) or in multiple (97.8%) infections. Single infections were equally distributed between sexes, while multiparasitisms were more likely to affect boys (63.0%, 29/46) than girls (36.9%, 17/46) (p = 0.021). Children of 5–9 years of age presented more frequently with single (62.2%, 56/90) and multiple (58.7%, 27/46) infections than children from any other age group considered in the study (p < 0.001).

**Table 3:** Distribution of protozoa, helminths and most frequent protozoa specie found in single- and multiple-infections, according to sex and age-group (N = total infected population; n = number of infected; % = percentage).

	Infection N = 136		Protozoa		*Blastocystis* sp.		Helminths	
	n	%	n	%	n	%	n	%
Total			126	92.6	49	38.9	19	13.9
Single	90	66.2	81	90	26	28.9	9	10
Sex								
Boys	38	42.2	34	89.5	14	36.8	4	10.5
Girls	52	57.8	47	90.4	12	23.1	5	9.6
Age group								
0–4	8	8.9	7	87.5	0	0	1	12.5
5–9	56	62.2	52	92.9	20	35.7	4	7.1
10–14	17	18.9	15	88.2	5	29.4	2	11.8
≥15	9	10	7	77.8	1	11.1	2	22.2
Multiple	46	33.8	45	97.8	23	50	10	21.7
Sex								
Boys	29	63	29	100	15	51.7	5	17.2
Girls	17	36.9	16	94.1	8	47.1	5	29.4
Age group								
0–4	6	13	6	100	1	16.7	0	0
5–9	27	58.7	26	96.3	13	48.1	7	25.9
10–14	13	28.3	13	100	9	69.2	3	23.1
≥15	0	0	0	0	0	0	0	0

*Blastocystis* sp. (38.9%, 49/126) was the most commonly distributed parasite species among infected schoolchildren. When single infections were considered, *Blastocystis* sp. carriage was predominantly (35.7%, 20/56) identified in children in the age group of 5–9 years (p = 0.043).

### 3.2 *Blastocystis* sp. subtyping

Out of the 49 stool samples that tested positive for *Blastocystis* sp. at microscopy examination, a total of 41 (83.7%) were available for downstream molecular analyses and yielded a positive result by *SSU*-PCR. Of them, 80.5% (33/41) were successfully subtyped by sequence analyses and 19.5% (8/41) were untypable due to insufficient quality sequence data. Typable isolates were unambiguously assigned to ST1 (36.4%), ST2 (21.2%), ST3 (39.4%), and ST1 + ST3 mixed infection (3.0%), with ST1 and ST3 being significantly (p = 0.002) more prevalent than ST2.

The frequency distribution of *Blastocystis* subtypes by sex and age group of the participating children is shown in Table 4. Among all successfully typed isolates, those characterized as ST1, ST2 and ST3 were significantly more frequent in children aged 5–9 years (p < 0.002, p = 0.005, p < 0.001, respectively). Boys were more likely to carry *Blastocystis* ST2 (p = 0.032), whereas ST3 was the only *Blastocystis* subtype detected in children younger than five years of age. One ST1 + ST3 mixed infection was found in a girl belonging to the 5–9 age group.

**Table 4:** Distribution of *Blastocystis* subtypes (ST) by sex and age group of the schoolchildren population from the surrounding urban area of Curitiba (Brazil) (N = total typable isolates of each ST; n = number of infected; % = percentage; 95% CI = 95% confidence interval; p-value = statistical value).

	ST1 N = 12				ST2 N = 7				ST3 N = 13				ST1 + ST3 N = 1			
	n	%	95% CI	p-Value	n	%	95% CI	p-Value	n	%	95% CI	p-Value	n	%	95% CI	p-Value
Sex																
Boys	7	58.3	30.2–82.8	0.683	6	85.7	46.9–99.3	0.032	7	53.8	27.4–78.7	0.999	0			–
Girls	5	41.7	17.2–69.8		1	14.3	0.7–53.0		6	46.2	21.3–72.6		1	100	5–100	
Age group																
0–4	0			<0.002	0			0.005	1	7.8	0.4–32.5	<0.001	0			–
5–9	9	75	45.9–93.2		5	71.4	33.0–94.9		9	69.2	41.3–89.4		1	100	5–100	
10–14	3	25	6.8–54.1		2	28.6	5.1–66.9		3	23.1	6.2–50.9		0			
≥15	0				0				0				0			

**Fig. 1:**
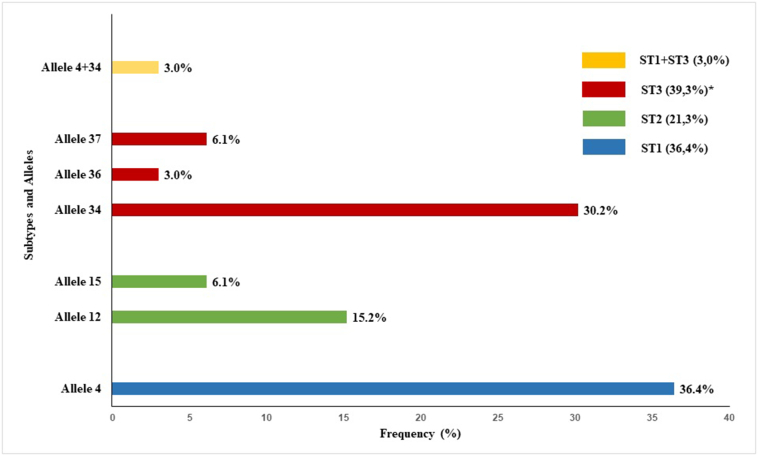
Diversity and frequency of *Blastocystis* subtypes and *SSU* rDNA dominant alleles identified in the schoolchildren population from the surrounding urban area of Curitiba (Brazil). Statistical significance (p < 0.01) is indicated by an asterisk.

Fig. 1 shows the distribution and frequencies of *Blastocystis* subtypes and *SSU* rDNA dominant alleles. According to their frequency of appearance, ST1 allele 4 (36.4%), ST3 allele 34 (30.2%) and ST2 allele 12 (15.2%) were more prevalent in the surveyed schoolchildren population. Additionally, and based on multiple sequence alignment analysis and chromatogram inspection, a mixed infection involving alleles 4 + 34 was identified in the *Blastocystis* ST1 + ST3 mixed infection sample.

## 4 Discussion

The overall infection rate (24.8%) obtained in the surrounding urban area of Curitiba is well in agreement with the gradual decreasing trend in the prevalence of human intestinal parasites reported in the Paraná state (Buschini et al., 2007: 75%; Seguí et al., 2018: 46%; Bosqui et al., 2015: 19%; Lopes et al., 2006: 18%; Santos and Merlini, 2010: 16%) during the last decades. Mass anthelminthic treatment campaigns and improved sanitary conditions implemented in Campo do Tenente during that period seemed to be extremely effective in reducing STH burden, as demonstrated by the low infection rate (3.5%) observed in the present study. This fact indicates a clear epidemiological shift towards an effective control of these diseases.

In contrast, even considering that only a single stool sample per participant was examined, the children population investigated here presented a high frequency of protozoa, both in single (90%) or multiple (97.8%) infections. The high prevalence of protozoan infections found in the area indicates that environmental contamination with human faeces is still a common event. Therefore, an interruption of ongoing anthelminthic treatment would negatively affect the campaign’s achievements, leading to an increased risk of re-infection by STHs.

Previous epidemiological studies conducted in the Paraná state also reported higher enteroparasites infection rates in urban rather than in rural areas (Valença Barbosa et al., 2017; Oliveira-Arbex et al., 2018; Cardoso et al., 2017; Ignacio et al., 2017; Bosqui et al., 2015; Lopes et al., 2006; Santos and Merlini, 2010; Moura et al., 2018). These findings are suggestive of a hyper-endemic situation within the municipality of Campo do Tenente, where urban slums are characterized by overcrowded population, lack of proper sanitation and reduced number of healthy housing, all factors driving the epidemic of protozoa infections. This situation is true even in areas considered as the most developed in the country, such as the south eastern and southern regions of Brazil (Buschini et al., 2007; Gil et al., 2013). The high level of protozoan infections observed in urban areas, including the downtown of the municipality of Campo do Tenente, demonstrate that there is still much to do to minimize the public health impact of these pathogens. Moreover, regular monitoring of the presence of protozoa transmitted by the faecal-oral route can be a useful indicator to gauge the performance of control campaign in place.

Urban slums are complex socio-environmental settings with a high degree of population vulnerability to infections. In addition to the infectious agents described above, we also detected several non-pathogenic protozoan species (*Entamoeba coli*, *Endolimax nana*, etc.) that are noteworthy because they share the same transmission pathways with pathogenic protozoa, and may be used as indicators of exposure to faecal contamination and sub-optimal hygienic practices.

More than half (66.2%) of the schoolchildren investigated here presented with single infections. Previous studies in the Paraná state reported that both single (Bosqui et al., 2015; Casavechia et al., 2016) and multiple (Seguí et al., 2018; Buschini et al., 2007) infections were a common finding in the human populations of the region. In other Brazilian states, multiparasitisms have been described in the range of 18.4% to 82% (Cardoso et al., 2017; Castro et al., 2015; Aguiar et al., 2007; Faria et al., 2017).

In our study, male and female schoolchildren had similar infection rates by intestinal parasites, with those in the age group of 5–9 years being particularly affected by these pathogens. Discrepant results between sexes have been reported in previous studies carried out in Brazil, with boys being found as infected as girls (Muniz et al., 2013), more infected than girls (Casavechia et al., 2016; Faria et al., 2017; Cabrine-Santos et al., 2015) or the opposite (Bosqui et al., 2015).

*Blastocystis* sp. was the most frequently found (38.9%) protist circulating in the investigated paediatric population. The occurrence of this parasite species has been identified at rates ranging from 1 to 33% in previous Brazilian studies (David et al., 2015; Cabrine-Santos et al., 2015; Basso et al., 2008; de Souza et al., 2007; Quadros et al., 2004). In the present survey complete socioeconomic, environmental, clinical and sanitary data were unavailable. Therefore, we could not go further in the epidemiological analysis of variables potentially associated with a higher risk of *Blastocystis* infection. However, our molecular and sequencing data allowed us to successfully subtype a total of 33 *Blastocystis*-positive samples, expanding our previous work in the Paranagúa Bay (Seguí et al., 2017, Seguí et al., 2018), and contributing to the current knowledge on the molecular epidemiology of this species in Brazil. We described the presence of three *Blastocystis* subtypes (ST1–3), with ST3 (39.4%) and ST1 (36.4%) being the most prevalent ones. Both STs have been reported previously in different Brazilian sates including Sao Paulo (David et al., 2015; Oliveira-Arbex et al., 2018; Melo et al., 2017) and Rio de Janeiro (Valença Barbosa et al., 2017, Valença Barbosa et al., 2018). Interestingly, a ST1 + ST3 mixed infection was identified in a low proportion (3.0%) of the investigated children, whereas other subtypes (e.g. ST4, ST6–8) were absent. In this regard, *Blastocystis* ST4 has been shown to have a marked geographical distribution, with most genotyped samples having a European origin. The relatively low genetic diversity of ST4 (evidence of a clonal population structure) suggests that ST4 may only have entered the human population recently, perhaps in Europe, and is yet to spread around the world through mass migration and international travel increase. This seems to be the situation in Brazil, where ST4 has been already identified at low rates in recent molecular epidemiological studies (Melo et al., 2017; Seguí et al., 2018). Subtypes ST5–9 have a more sporadic occurrence in humans and are probably the result of zoonotic events (Stensvold et al., 2009, Stensvold et al., 2007, Stensvold et al., 2008). Human infection by ST6 and ST7 appears to be more common in certain countries, such as Japan (Kaneda et al., 2001).

Finally, ST3 was more prevalently found in girls than in boys, and was also the only subtype identified in children younger than five years of age. These results are in contrast with previous work in Paraná state where ST3 was more frequently detected in males younger than 10 years-old (Seguí et al., 2018). These data may be indicative of distinct *Blastocystis* transmission patterns that remain to be fully elucidated. In this regard, the absence of *Blastocystis* subtypes other than STs 1–4, known to be frequent in non-human animal species, support the idea that *Blastocystis* transmission in the urban slums of the Campo do Tenente municipality is primarily anthroponotic in origin.

## 5 Conclusion

The low prevalence of STHs (3.5%) detected in the surrounding urban area of Curitiba, in contrast with the high protozoan frequency both in single (90%) and in multiple (97.8%) infections, provides evidence of the effectiveness of the anthelminthic treatment campaigns implemented in the region. These data also highlight the convenience of conducting periodical coproparasitological analysis as a practical option to assess the impact and success of control campaigns in deprived urban areas. It seems clear now that it is time to take action against diarrhoea-causing protozoan infections by implementing effective water, sanitation, hygiene, and education interventions to minimize the risk of environmental contamination with human excreta, in order to improve the standards of living of people in Brazilian urban communities and neighbourhoods.

## Declaration of competing interest

None.
